# Cervical neurofibromatosis with quadriparesis: Management by fibular strut graft

**DOI:** 10.4103/0019-5413.54968

**Published:** 2010

**Authors:** Wichien Laohacharoensombat, Wiwat Wajanavisit, Patarawan Woratanarat

**Affiliations:** Department of Orthopaedics, Ramathibodi Hospital, Mahidol University, Bangkok 10400, Thailand

**Keywords:** Cervical kyphosis, cervical spondyloptosis, cervical neurofibromatosis, fibular strut graft

## Abstract

This is a case report of an eight-year old boy with neurofibromatosis and a 120° dystrophic kyphosis of the cervical spine. He presented with progressive quadriparesis caused by spondyloptosis of the C2/C3, and was successfully treated by skull traction and one-stage anterior fibular strut graft lying between the tubercle of the atlas through the C2 body slot and lower vertebrae. At seven years follow-up there was, loosening of lower vertebral screws which allowed growth and residual mobility of lower vertebral joints while the fusion of upper cervical spines was still solid.

## INTRODUCTION

Paraplegia is a common finding in neurofibromatosis with dysplastic deformity. Dysplastic deformity is usually found in thoracic and lumbar spines.^1^–^5^ In only rare cases, dystrophic neurofibromatosis type I may involve upper cervical spines leading to severe kyphoscoliosis endangering the spinal cord.^6^,^7^ Previous reported cases of severe kyphosis and myelopathy of the cervical spines were treated by one-stage anterior and posterior procedures.^8^–^10^ We present a boy with classical neurofibromatosis, with an extreme degree of kyphosis and spondyloptosis of the upper cervical spines, treated by one-stage anterior autogenous fibular strut graft inserted through the anterior slot of axis to support the tubercle of atlas.

## CASE REPORT

This eight-year-old boy presented with the problem of inability to walk for one week. A large café-au-lait spot was detected on the right side of his neck since birth. A growing soft tissue mass had been found on right side of the neck since he was one year old. Despite experiencing the mass and neck deformity, his daily life, during childhood, was normal including attending the local primary school. Four months prior to admission, he developed progressive weakness of all extremities. Bowel and bladder control were normal. No relevent family history was found. At the first examination, in August 2000, a large neurofibroma of 15 centimeters in diameter was noted on the right side of his neck, extending from the right shoulder to the right ear. The head was tilted forward and laterally to the left side. He could not tilt his neck to the right side.

**Figure 1:**
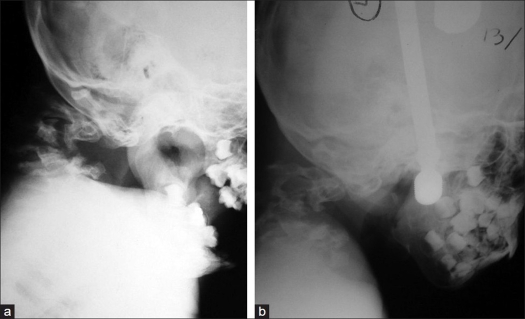
A 8-year-old boy presented with cervical kyphosis and inability to walk. Lateral radiograph of cervical spine (a) shows spondyloloptosis at C2/C3 with kyphotic angle between C2/4 of 120°. After skull traction, radiograph (b) shows some degree of kyphotic correction

**Figure 2:**
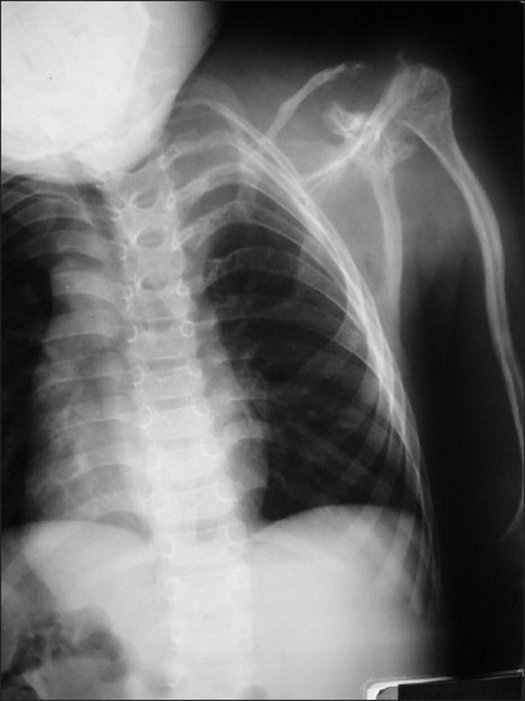
The radiograph shows dysplastic left clavicle, humerus and the first four ribs

**Figure 3:**
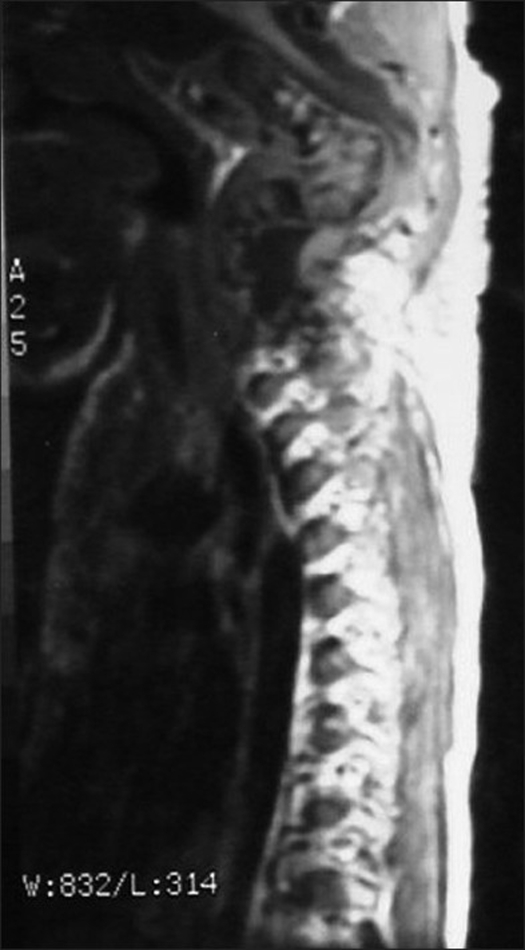
The magnetic resonance imaging (T_2_W sagital view) shows the stretched out cervical cord that was compressed at the apex of the kyphosis

**Figure 4:**
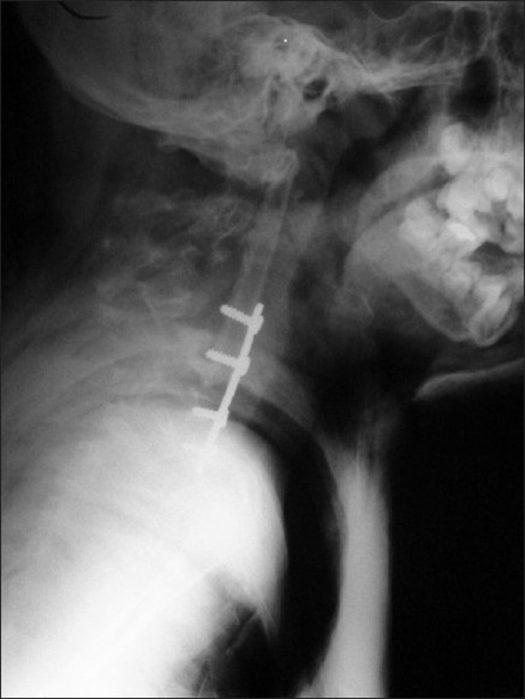
After surgery, the cast was maintained for 4 months. The radiograph show the evidence of bony healing

Neurological examination revealed weakness on all extremities (left side grade 2-3/5, right side grade 3-4/5) with obvious long tract signs. Radiological examination revealed spondyloloptosis at C2/C3 with kyphotic angle between C2/4 of 120° [Figure 1]. The left clavicle, humerus, and the first four ribs were found to be dysplastic [Figure 2]. Mild scoliosis of the thoracic spine was noted. The magnetic resonance scan of the C-spines showed a spondyloptotic-kyphotic deformity of the spine of more than 120° at C2-C4 vertebral levels. The cervical cord was stretched and compressed at the apex of the kyphosis [Figure 3]. Mild-to-moderate cord atrophy was noted. Dural ectasia of upper cervical spine was also detected. Skull traction with one-kilogram weight was applied on the second day of admission. The motor power of all extremities improved one grade on the third day. The kyphotic angle was improved to 90°. The patient underwent surgery on the 12^th^ day of admission. The skull traction was maintained during intra- and post-operative period. The left anterior para-sternocleidomastoid incision was used, extending from the sub-mandibular to supra-clavicular region. After blunt dissection, the lower part of vertebral bodies, C2 to C7, were exposed. Discectomy of the C3/C4 disc facilitated the removal of C3 body. The position of the spine was re-adjusted to reduce kypho-scoliosis deformity under 5 SEP-monitoring. A small slot was created at the anterior port of C2, and C4 – C7 bodies. A 10-centimeter fibular graft was taken sub-periosteally from the middle-third of left leg. Its cephalous end was embedded in the C2 slot and was then hammered proximally to support tubercle of the atlas. The caudal end of the graft was subsequently pushed into the slot of lower vertebral bodies (C4-C7). Four lag screws were used to fix the distal end of fibular graft to the C4-C7 bodies respectively. Post-operatively, the neurological status of the patient was stable. The kyphotic angle between C2-C4 was reduced to 55°. The traction was securely maintained for one week. A Minerva cast was applied on the 10^th^ day after surgery. The patient was discharged two weeks after surgery. The cast was maintained for four months and removed when bone healing was roengentnographically evident [Figure 4]. The neurological status gradually improved.

**Figure 5:**
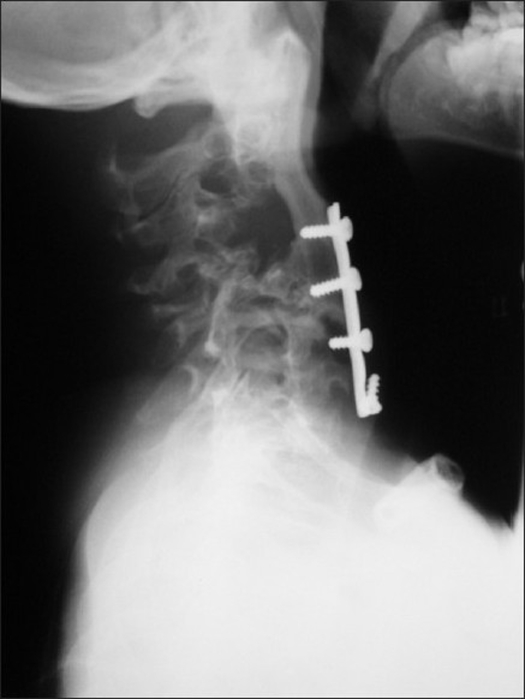
The radiograph shows the lower 2 screws were loosened during the growth of the lower cervical spines (C6 and C7)

The patient was independent by two months of post-operative period and normal activity was achieved by six months. Further remodeling of the graft was completed in three years. The latest kyphotic angle was 50°. The lower two screws were loosened in accordance with bone growth. Cervical spines (C6 and C7) regained its growth and mobility [Figure 5]. The patient can spend his normal life at the last follow up (six years post-operatively) by attending a regular class.

## DISCUSSION

Dystrophic neurofibromatosis type I, involving upper cervical spines, is rare. When it appears, it can lead to serious neurological complications. Surgical treatment has inherent risks and difficulties due to several reasons. Firstly, poor bone quality is prone to difficult rigid fixation. Secondly, the anterior approach to upper cervical region is not very feasible even if we disregard bizarre deformities. Thirdly, manipulation of the extreme degree of deformity in the presence of compromised cord may lead to more cord damages and ischemia.

Some authors suggested using combined anterior and posterior approach, such as Kokubun *et al*. (1992)^8^ and Gottin and Dieter (1999).^9^ The children in those reports were treated by anterior grafting up to C2 level. Since the apex of kyphosis in our case was at the C2/C3 level, the fusion had been extended to C1 to achieve a stable fusion mass in the remnant kyphotic deformity. We propose the technique of partial embedding of the upper end of the fibula into the slot of C2 body so that the part of the upper end of the graft can support the tubercle of C1. To prevent tilting of fibula graft, the lower end of the graft was fixed to the vertebral bodies with screws. The graft would also prevent progression of kyphosis during healing period.

At three years of follow-up, the graft remodeled nicely with obvious stability of the fused segments. Loosening of screws at the lower end of the graft was partly due to the growth of lower vertebral bodies. Fortunately, this would allow some remnant of mobility. The patient refused to have the metals removed as he felt there was no problem. The results from our study confirm the high potential of remodeling of the cervical deformities proposed by to Shiaki *et al*.^10^
